# A proposed index of myocardial staining for vein of Marshall ethanol infusion: an Italian single-center experience

**DOI:** 10.1007/s10840-023-01732-4

**Published:** 2024-01-11

**Authors:** Federico Landra, Martina Nesti, Silvia Garibaldi, Gianluca Mirizzi, Umberto Startari, Luca Panchetti, Marcello Piacenti, Simone Taddeucci, Bruno Antonio Formichi, Maurizio Stefani, Serena Galiberti, Vincenzo Lionetti, Paolo Solinas, Beatrice Maria Levantesi, Chiara Italia, Andrea Rossi

**Affiliations:** 1 Division of Cardiology, Department of Medical Biotechnologies, University of Siena, Viale Bracci 1, Siena, Italy; 2 Fondazione Toscana Gabriele Monasterio, Pisa, Italy

**Keywords:** Persistent atrial fibrillation, Staining, Mitral isthmus, Vein of Marshall, Ethanol infusion, Catheter ablation

## Abstract

**Background:**

Mitral isthmus (MI) conduction block is a fundamental step in anatomical approach treatment for persistent atrial fibrillation (PeAF). However, MI block is hardly achievable with endocardial ablation only. Retrograde ethanol infusion (EI) into the vein of Marshall (VOM) facilitates MI block. Fluorographic myocardial staining (MS) during VOM-EI could be helpful in predicting procedural alcoholization outcome even if its role is qualitatively assessed in the routine. The aim was to quantitatively assess MS during VOM-EI and to evaluate its association with MI block achievement.

**Methods:**

Consecutive patients undergoing catheter ablation for PeAF at Fondazione Toscana Gabriele Monasterio (Pisa, Italy) from February 2022 to May 2023 were considered. Patients with identifiable VOM were included. A proposed index of MS (MSI) was retrospectively calculated in each included patient. Correlation of MSI with low-voltage zones (LVZ) extension after VOM-EI and its association with MI block achievement were assessed.

**Results:**

In total, 42 patients out of 49 (85.8%) had an identifiable VOM. MI block was successfully achieved in 35 patients out of 42 (83.3%). MSI was significantly associated with the occurrence of MI block (OR 1.24 (1.03–1.48); *p* = 0.022). A higher MSI resulted in reduced ablation time (*p* = 0.014) and reduced radiofrequency applications (*p* = 0.002) to obtain MI block. MSI was also associated with MI block obtained by endocardial ablation only (OR 1.07 (1.02–1.13); *p* = 0.002). MSI was highly correlated with newly formed LVZ extension (*r* = 0.776; *p* = 0.001).

**Conclusions:**

In our study cohort, optimal MSI predicts MI block and facilitates its achievement with endocardial ablation only.

## Introduction

Recent technological advances have raised the efficacy of pulmonary vein isolation (PVI) even in patients with persistent atrial fibrillation (PeAF) [1]. However, a higher arrhythmogenesis complexity in PeAF has yielded a variety of additional treatment strategies to PVI in the last years, aiming at either substrate modification or trigger modulation [2, 3].

Mitral isthmus (MI) conduction block validation is a fundamental step in perimitral flutter ablation and anatomical approach treatment for persistent atrial fibrillation [3, 4]. However, MI block is rarely achievable with endocardial radiofrequency ablation only [5]. The vein of Marshall (VOM) is anatomically linked to MI and its epicardial musculature may prevent its complete block [6–9]. Also, VOM has been recognized as an important factor for AF initiation and maintenance, making it an appealing target for ablation procedures [6, 10, 11].

Ethanol infusion into the VOM (VOM-EI) facilitates MI block by targeting its epicardial muscular bundles and ablating related portions of left atrial (LA) myocardium [7, 12–15]. Previous studies have reported the efficacy and safety of VOM-EI [16–18]. A comprehensive anatomical approach to PeAF ablation including VOM-EI has proven to be feasible, safe, and associated with a high rate of freedom from arrhythmia recurrences [19].

Optimal myocardial staining (MS) occurring during VOM-EI is traditionally considered a marker of efficacy and used as a guidance during EI, even though the presence of localized staining has not been correlated to the clinical outcome [20]. However, its value could be limited by the qualitative nature of the assessment.

The aim of this study is to provide insights into the potential role of MS in predicting MI block achievement during PeAF ablation.

## Methods

### Patient selection

Consecutive patients undergoing catheter ablation for PeAF at Fondazione Toscana Gabriele Monasterio (Pisa, Italy) from February 2022 to May 2023 were considered. Patients with identifiable VOM were prospectively included in the registry and data were collected on a dedicated database. Patients were excluded if under 18 years of age or did not provide informed consent and if they underwent previous AF ablation procedures without PVI only. All procedures were performed under general anesthesia.

### Electroanatomical voltage mapping

**Fig. 1:**
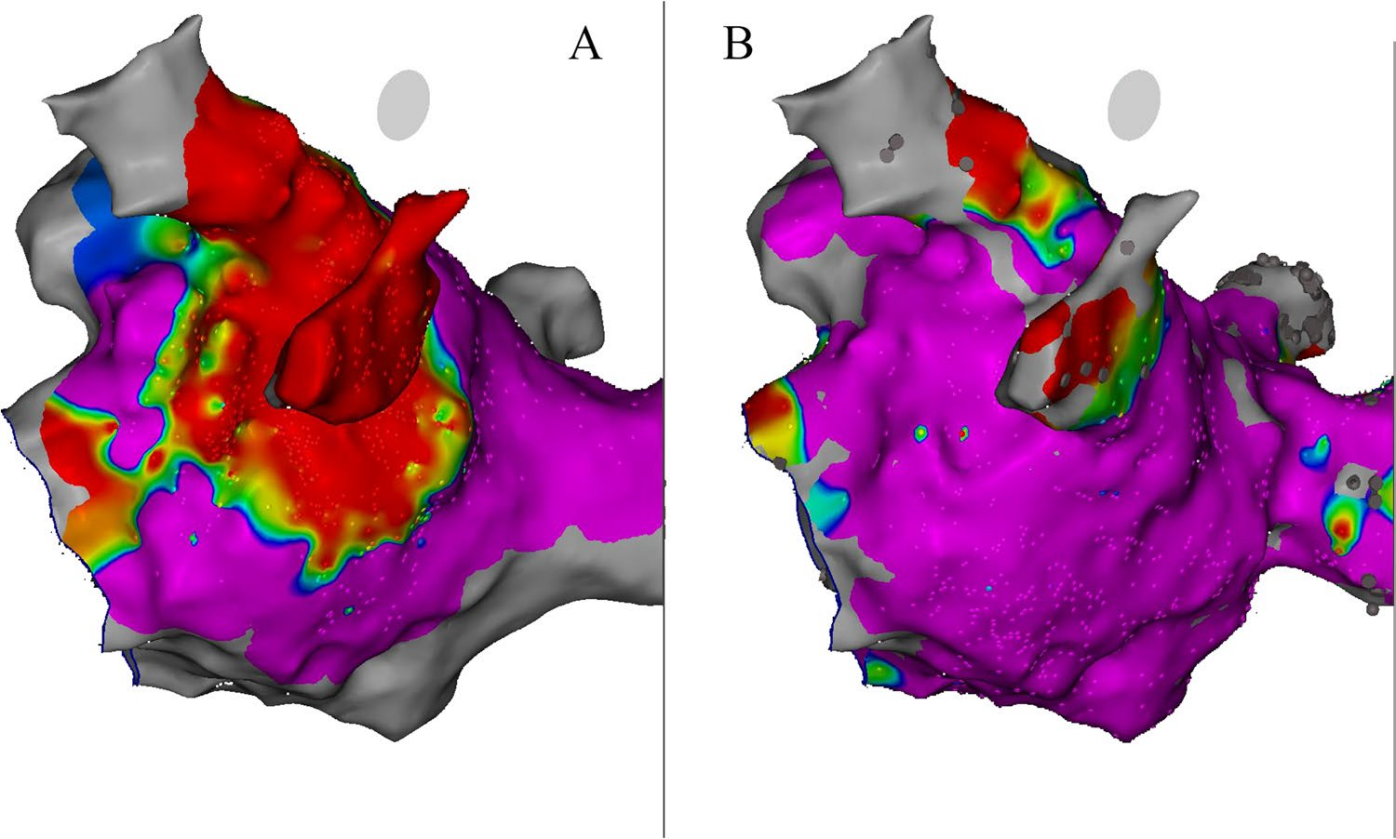
Low-voltage zones at mitral isthmus after (**A**) and before (**B**) vein of Marshall ethanol infusion. Note the collar-shaped lesion around left pulmonary veins. Only a subtle gap towards mitral annulus remains to achieve mitral isthmus block

Catheter ablation procedures were performed using either CARTO 3® (Biosense Webster, Inc.) or EnSite X® (Abbott, Inc.) 3D mapping systems. High-density bipolar voltage mapping at MI level with either PentaRay™ or Advisor HD Grid™ catheters was performed before and immediately after VOM-EI. Bipolar map cutoffs were set to 0.05–0.50 mV in case of sinus rhythm or 0.05–0.29 mV in case of atrial fibrillation. Low-voltage zones (LVZs) at MI before and after VOM-EI were calculated in post-process always by the same operator blinded to procedural results (Fig. 1).

### Ethanol infusion in the vein of Marshall

VOM-EI was performed before catheter ablation. Coronary sinus cannulation was obtained with either non-steerable (Swartz SL0, Abbott, Inc.) or steerable introducers (Agilis NxT, Abbott, Inc.). VOM was initially searched by coronary sinus venography and therefore selectively cannulated using a left internal mammary artery (LIMA) diagnostic catheter. A 0.014-in. Balance Middleweight Universal Guide Wire and an over-the-wire (OTW) angioplasty balloon (Emerge, Boston Scientific, 1.5–2.0–2.5 × 8–12 mm) were used to occlude the VOM at its ostium. Selective VOM venography throughout the OTW balloon was performed to confirm position. VOM-EI was then performed: a total of 10 cc of ethanol was infused in 10 min in slow boluses of 3 or 4 cc. Slow injections are needed in order to prevent VOM dissection and perforation. After each bolus of ethanol 1 cc of medium contrast was injected to check the MS, and verify the absence of VOM dissection/perforation or extravasation of contrast medium in the pericardial space. Intracardiac echocardiography was used to assist the procedure.

### Catheter ablation

Catheter ablation was subsequently performed with the use of radiofrequency energy delivered by QDOT MICRO™ or Smarttouch ST/SF™ (Biosense Webster, Inc.) or TactiCath™ (Abbott, Inc.) ablation catheters. De novo patients underwent wide antral PVI, while all patients underwent mitral, roof, and cavotricuspid isthmuses linear lesions [19]. MI ablation line was performed with point-by-point radiofrequency delivered with an interlesion distance of 4 mm, for 15–20 s with a power output of 40–45 W, with temperature limited to 45°C, and normal saline irrigation (8–20 mL/min). MI line was performed during left atrial appendage (LAA) pacing at 600-ms pacing cycle length. In the presence of residual epicardial connection across MI, additional radiofrequency ablations were performed at the great cardiac vein (GCV) level, either at the anchoring or free-wall side, for 20 s with a power output of 20–25 W, as previously described [5]. All linear lesions required confirmation of bidirectional conduction block by differential pacing.

### Myocardial staining index

**Fig. 2:**
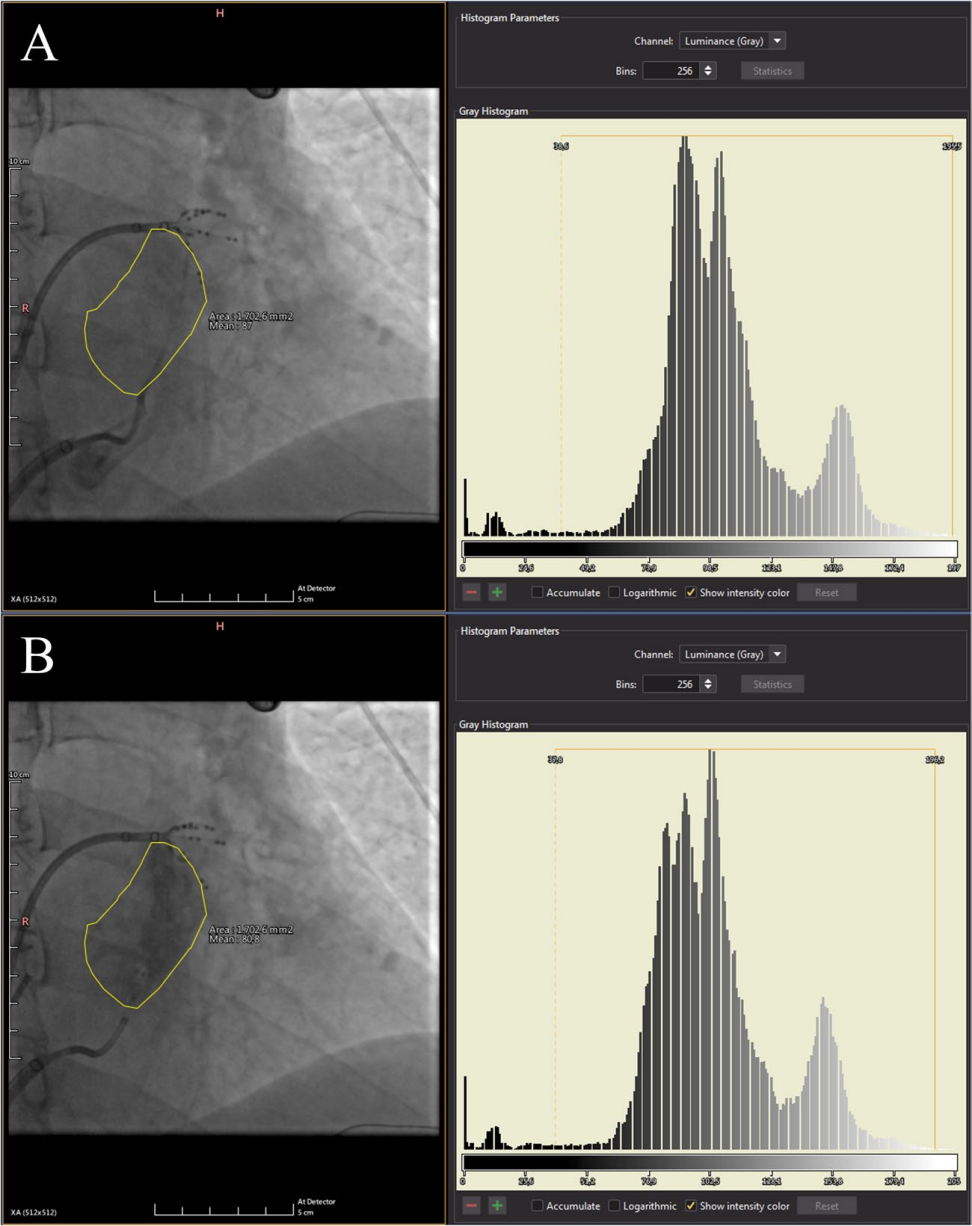
Fluorographic right anterior oblique 30° projection was used to encircle myocardial staining area (**B**). The same area was applied to the same fluorographic acquisition prior to vein of Marshall ethanol infusion (**A**) in order to calculate only the additional opacity brought by myocardial staining. This is the same patient as in Fig. 1. Optimal myocardial staining predicted ease of mitral isthmus block achievement with endocardial ablation only. On the right, relative pre- and post-ethanol infusion histograms of luminescence

The proposed index to quantitatively assess MS after VOM-EI was retrospectively calculated using fluorographic acquisitions post-processed with Weasis DICOM medical viewer software v4.0.2 (University Hospital of Geneva, Switzerland). Analysis was performed by the same operator (F.L.) blinded to procedural results. A right anterior oblique (RAO) 30° without caudo-cranial angle fluorography was used. The area of MS was calculated by encircling the whole area penetrated by the contrast medium at the end of the VOM-EI protocol. The myocardial staining index (MSI) was calculated from the product of the area of MS and the delta fluorographic luminescence of the area of interest between pre- and post-VOM-EI and expressed in cm^2^ × Gray (Fig. 2). It did not appear to be any difference on final MSI according to speed or pressure of contrast injection, as long as the VOM remained intact.

### Follow-up

Latest available follow-up was performed by means of implantable loop recorders (ILR), cardiac implanted electronic devices (CIED), or periodic 24-h Holter monitoring at 1, 6, 12, and 18 months from index procedure. Each ILR/CIED-detected arrhythmic event was carefully reviewed by a blinded board-certified cardiologist to account for potential overdiagnoses. A 1-month blanking period was considered [22]. Continuation of antiarrhythmic drugs (AADs) after the blanking period was at operator’s discretion and was recorded. Arrhythmic recurrences were defined as episodes of atrial tachycardia/AF lasting more than 30 s [22]. In patients with ILR and CIED, number of episodes, maximum duration, and arrhythmic burden were recorded as well.

### Statistical analysis

Continuous data are presented as mean and standard deviation or as median and interquartile range, as appropriate. Kolmogorov–Smirnov test was used to verify normal distribution of variables. Categorical data are summarized as absolute and relative frequencies. Continuous variables were compared using the unpaired *t* test for normally distributed variables and the nonparametric Mann–Whitney *U* test for non-normally distributed variables. The Chi-square test was used for categorical variables. Correlation was calculated using Pearson’s correlation coefficient. Univariate regression analysis was performed to calculate the association between MSI, delta LVZs, and MI block. Receiver operating characteristic (ROC) curves were generated to assess predictive performance of MSI and delta LVZs for MI block and of MSI for arrhythmia recurrences. The Youden index was used to find the best cutoff. Kaplan–Meier analysis and Cox proportional hazard models were performed to evaluate cumulative event rates at follow-up and results are presented as hazard ratios (HR) and 95% confidence intervals (CI). In case of missing data, no statistical method for imputation was performed. A *p*-value < 0.05 was considered statistically significant. Analysis was performed using SPSS, version 26 (SPSS, Chicago, IL, USA), and R Statistical Software, version 3.6.3 (R Foundation for Statistical Computing, Vienna, Austria).

## Results

### Patient population

Between February 2022 and May 2023, 49 patients were scheduled for VOM-EI at Fondazione Toscana Gabriele Monasterio (Pisa, Italy) for PeAF ablation. In seven out of 49 patients (14.2%), VOM was not present. A total of 42 consecutive patients were prospectively included in the registry. Nine patients (21.4%) had early PeAF (< 3 months), 21 patients (50%) had PeAF, and 12 patients (28.6%) had long-standing PeAF (> 12 months). Thirteen patients (30.9%) were redo cases and previously underwent PVI only. For complete baseline characteristics, see Table 1. None of the baseline variables significantly differed between the two groups of patients divided for MI block achievement.

**Table 1:** Baseline characteristics

	All patients (*n* = 42)	Mitral block achieved (*n* = 35)	Mitral block not achieved (*n* = 7)	*p*-value
Male	29 (69.0%)	25 (71.4%)	4 (57.1%)	0.455
Age (years)	66 ± 7	66 ± 7	65 ± 6	0.648
Hypertension	26 (61.9%)	23 (65.7%)	3 (42.9%)	0.256
Diabetes	4 (9.5%)	4 (11.4%)	0 (0%)	0.347
Obesity	10 (23.8%)	9 (25.7%)	1 (14.3%)	0.517
CHA_2_DS_2_-VASc	2 (1 – 3)	2 (1 – 3)	2 (1 – 3)	0.817
CHF	9 (21.4%)	8 (22.9%)	1 (14.3%)	0.614
Stroke	6 (14.3%)	5 (14.3%)	1 (14.3%)	1.000
Years from first AF episode	4 (2 – 8)	4 (2 – 8)	3 (2 – 7)	0.741
Previous ablation	13 (30.9%)	12 (34.3%)	1 (14.3%)	0.296
Type of AF				
Early persistent	9 (21.4%)	8 (22.9%)	1 (14.3%)	0.614
Persistent	21 (50.0%)	18 (51.4%)	3 (42.9%)	0.679
Long-lasting persistent	12 (28.6%)	9 (25.7%)	3 (42.9%)	0.359
LA volume (ml/m^2^)	34 (28 – 41)	34 (29 – 41)	33 (24 – 51)	0.766
LVEF (%)	61 (55 – 65)	62 (55 – 65)	57 (55 – 70)	0.895

*CHF*, congestive heart failure; *AF*, atrial fibrillation; *LA*, left atrium; *LVEF*, left ventricular ejection fraction

### Procedural data

Thirty-one patients were in sinus rhythm (73.8%), and 11 were in atrial fibrillation (26.2%). LVZs at MI were observed in 14 patients (33.3%). Median VOM length was 42.1 mm (IQR 37.6–58.1 mm). Overall, 23 patients (54.8%) received endocardial MI ablation only, while 19 patients (45.2%) also received anchoring-GCV ablation, and 10 patients (23.8%) free-wall GCV ablation. MI block was successfully achieved in 35 patients out of 42 (83.3%) (Table 2). VOM perforation occurred in three patients (7.1%), in two cases after the first injection, and in one case after the second one. Coronary sinus dissection occurred in one patient (2.4%). Only one case of VOM perforation resulted in acute pericarditis, with occurrence of pericardial effusion but not tamponade. Considering them as separate events, the overall rate of complication was 11.9% (five events in 42 patients). Even though MSI was calculated only using the fluorography acquired at the end of VOM-EI, in our experience MS showed to progressively increase with each ethanol injection in a linear fashion, as long as VOM remained intact.

**Table 2:** Procedural data

	All patients (*n* = 42)	Mitral block achieved (*n* = 35)	Mitral block not achieved (*n* = 7)	*p*-value
Baseline rhythm				
Sinus	31 (73.8%)	27 (77.1%)	4 (57.1%)	0.272
AF	11 (26.2%)	8 (22.9%)	3 (42.3%)	0.293
VOM length (mm)	42.1 (37.6 – 58.1)	47.5 (38.6 – 58.1)	28.5 (28.1 – 28.6)	0.398
Staining area (cm^2^)	8.63 ± 3.86	9.16 ± 3.76	4.97 ± 2.51	0.021
Delta fluorographic luminescence (Gray)	6.0 ± 2.9	6.4 ± 2.8	3.1 ± 1.6	0.013
MSI (cm^2^ × Gray)	43.8 (26.1 – 57.7)	48.8 (38.3 – 59.2)	13.3 (2.3 – 22.1)	< 0.001
Localized staining	4 (14.3%)	3 (8.6%)	1 (14.3%)	0.349
LVZs pre (cm^2^)	0 (0 – 2.9)	0 (0 – 1.5)	2.8 (0 – 4.5)	0.109
LVZs post (cm^2^)	7.6 (4.8 – 11.9)	8.4 (5.0 – 12.0)	7.0 (1.0 – 7.6)	0.100
Delta LVZs (cm^2^)	6.3 ±3.8	7.2 ± 3.5	1.9 ± 1.8	< 0.001
Ablation time (s)	2081 ± 2034	1459 ± 1788	3636 ± 1955	0.068
RF applications (#)	26 (15 – 44)	24 (11 – 30)	95 (51 – 165)	0.002
Endocardial ablation only	23 (54.8%)	23 (65.7%)	n.a	n.a
Anchoring GCV	19 (45.2%)	12 (34.3%)	7 (100%)	0.001
Free-wall GCV	10 (23.8%)	3 (8.6%)	7 (100%)	< 0.001

*AF*, atrial fibrillation; *VOM*, vein of Marshall; *MSI*, myocardial staining index; *LVZs*, low-voltage zones; *RF*, radiofrequency; *GCV*, great cardiac vein

### Association of MSI and LVZs with mitral isthmus block

**Fig. 3:**
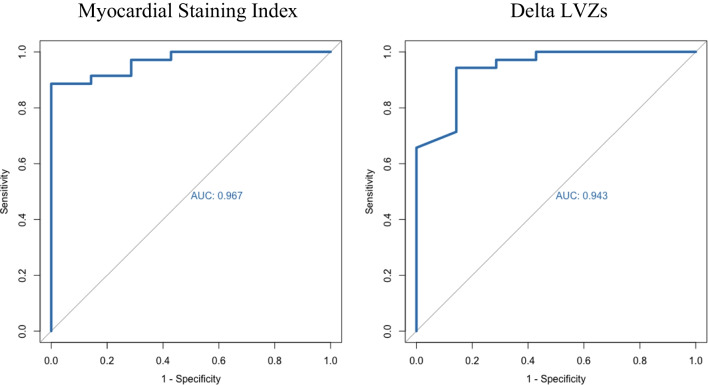
Receiver operating characteristics curves for prediction of mitral isthmus block for myocardial staining index (on the left) and delta LVZs (on the right). LVZs, low-voltage zones; AUC, area under the curve

**Fig. 4:**
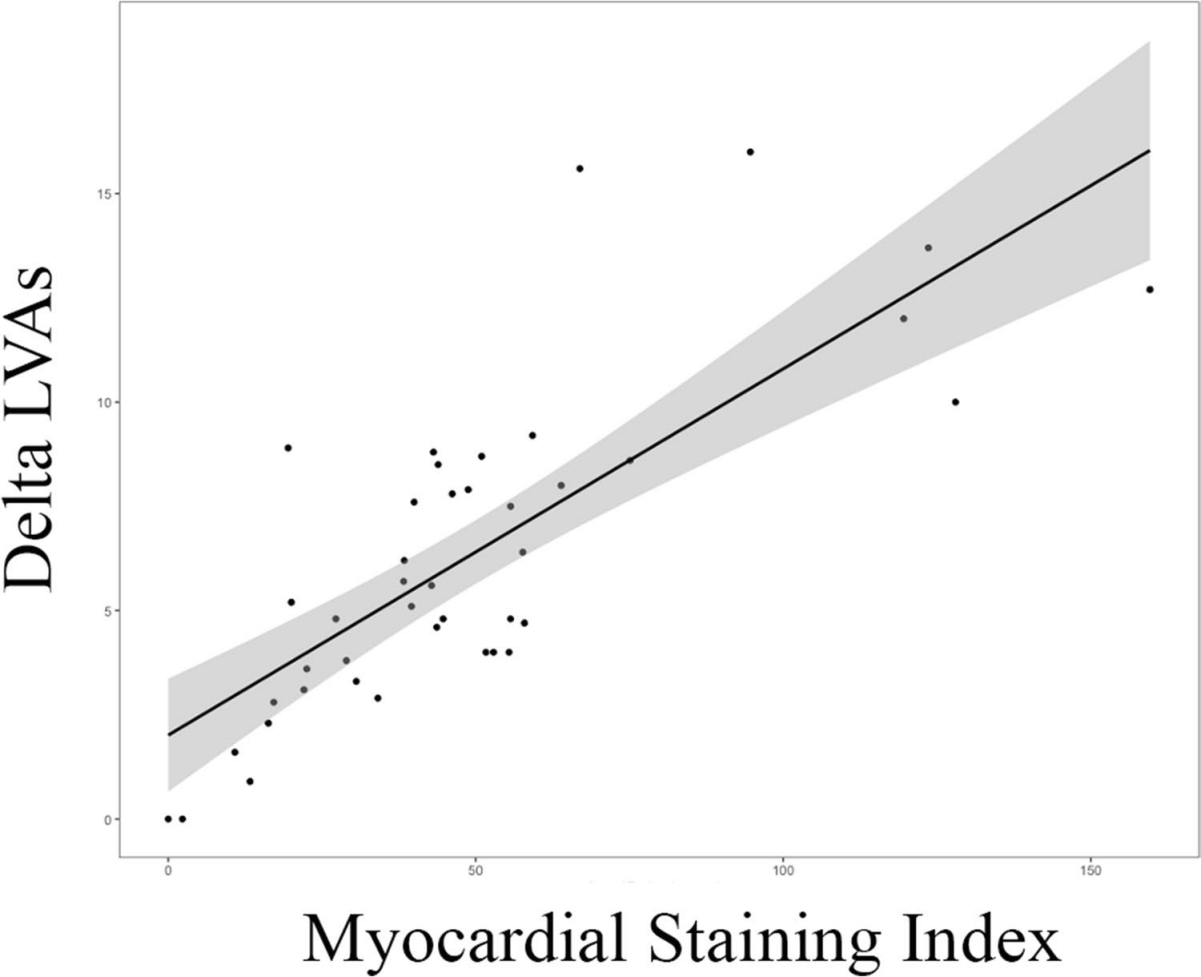
Correlation between myocardial staining index and delta LVZs. LVZs, low-voltage zones

**Fig. 5:**
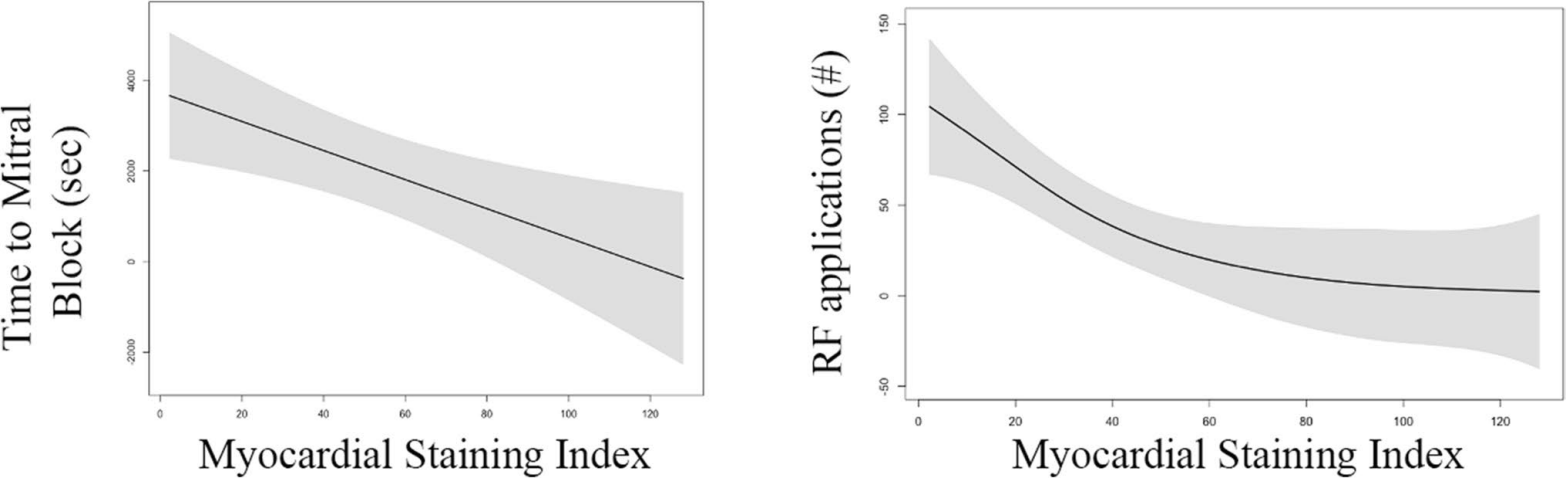
Correlation between myocardial staining index and ablation time to obtain mitral isthmus block (on the left) and number of radiofrequency applications (on the right). RF, radiofrequency

Significant differences were observed between the two groups of patients according to MI block achievement regarding delta LVZs, staining area, delta fluorographic luminescence, and MSI (*p* < 0.05). Occurrence of localized staining did not differ between the two groups (*p* = 0.349). At univariate regression analysis, delta LVZs (OR 3.82 (1.31–11.16); *p* = 0.014), staining area (OR 1.01 (1.00–1.01); *p* = 0.036), delta fluorographic luminescence (OR 2.64 (1.08–6.46); *p* = 0.034), and MSI (OR 1.24 (1.03–1.48); *p* = 0.022) confirmed to be significantly associated with the occurrence of MI block. ROC curves showed high predictive performance of MSI (AUC = 0.967) and delta LVZs (AUC = 0.943) for MI block (Fig. 3). MSI and delta LVZs were highly correlated variables (*r* = 0.776; *p* = 0.001) (Fig. 4). Importantly, higher MSI resulted in reduced ablation time (*p* = 0.014) and reduced RF applications (*p* = 0.002) at MI (either endocardial or at GCV) to obtain MI block (Fig. 5). Moreover, MSI was significantly associated not only with the occurrence of MI block, but also with its achievement by endocardial ablation only (OR 1.07 (1.02–1.13); *p* = 0.002). Indeed, MSI was significantly higher (*p* < 0.001) in those who required endocardial ablation only (55.4 cm^2^ × Gray (IQR 43.7 – 75.1 cm^2^ × Gray)) in contrast to those who needed additional GCV ablation (22.5 cm^2^ × Gray (IQR 16.3 – 43.1 cm^2^ × Gray)). Finally, MSI was lower in patients who experienced VOM perforation as compared to the others [10.8 (2.3 – 20.0) vs. 44.7 (30.6 – 57.9), *p* = 0.005] and it was not calculable in the patient who experienced coronary sinus dissection, because it happened prior to VOM cannulation. Only one out of four patients (25%) who either experienced VOM perforation or coronary sinus dissection eventually reached MI block.

### Follow-up

ILR were implanted at the end of the procedure in each patient without prior ablation procedures (29 out of 42 patients, 69.1%) and in one patient out of 13 (7.7%) of those with prior ablation procedures. Three patients out of 42 (7.1%) had already a CIED. Mean follow-up was 249 ± 126 days. Overall, in 28 patients out of 42 (66.7%) AADs were discontinued after 1 month from the procedure. AADs were more frequently discontinued in patients naïve from prior ablation procedures (21 out of 29 patients, 72.4%) as compared to redo cases (7 out of 13 patients, 53.8%), even though not significantly (*p* = 0.238). Overall, 32 patients out of 42 (76.2%) were free from arrhythmia recurrences at latest available follow-up, of which 24 patients were naïve patients (24 patients out of 29, 82.3%) and eight patients were redo cases (8 patients out of 13, 61.5%) (*p* = 0.136). Four patients had AF recurrence (four out of 10 patients, 40.0%), five patients had a recurrence as atrial flutter (five out of 10, 50.0%), and one patient had both AF and atrial flutter recurrences (one out of 10, 10%). Among them, six out of 10 patients had either an ILR or CIED, which showed a mean burden of 0.64 ± 0.81%, a maximum of 17 episodes/patient and < 24-h duration.

### Association of MSI with arrhythmia recurrence

**Fig. 6:**
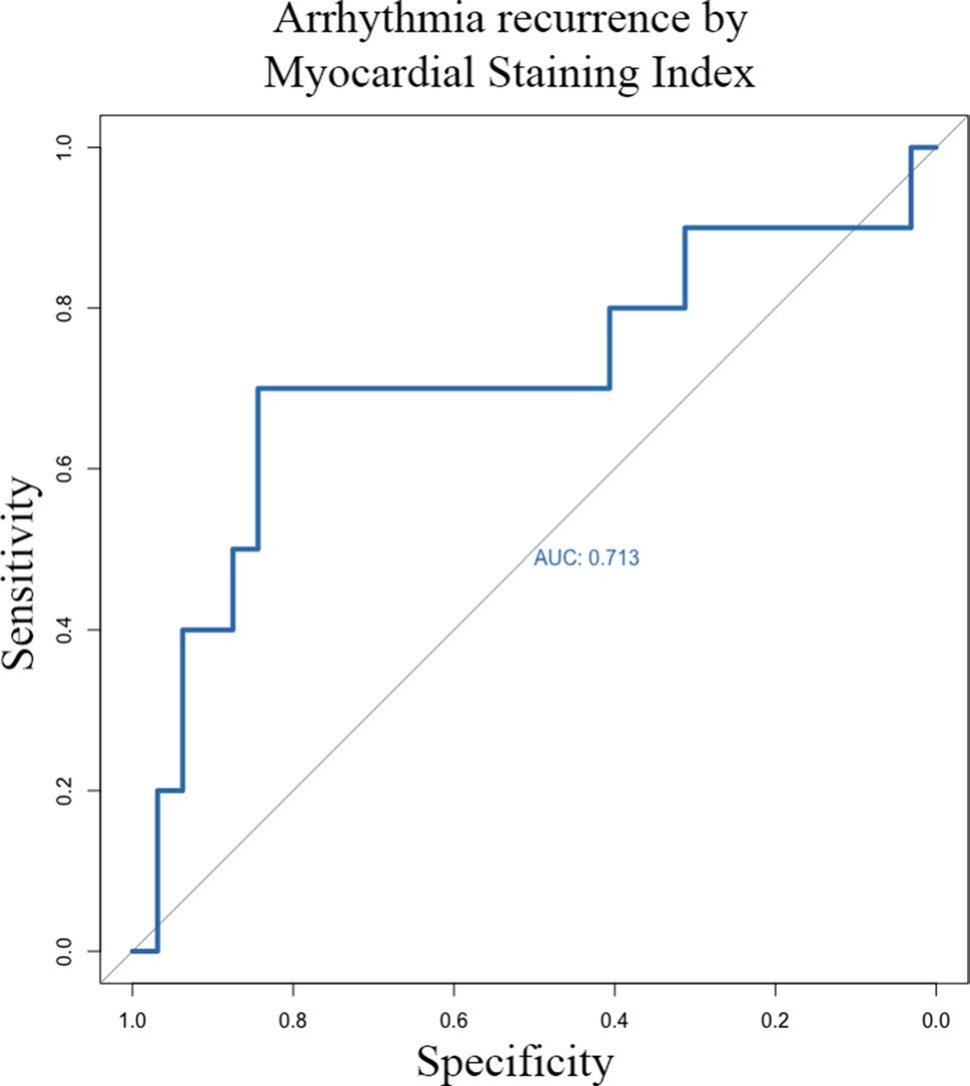
Receiver operating characteristics curves for prediction of arrhythmia recurrence for myocardial staining index. AUC, area under the curve

**Fig. 7:**
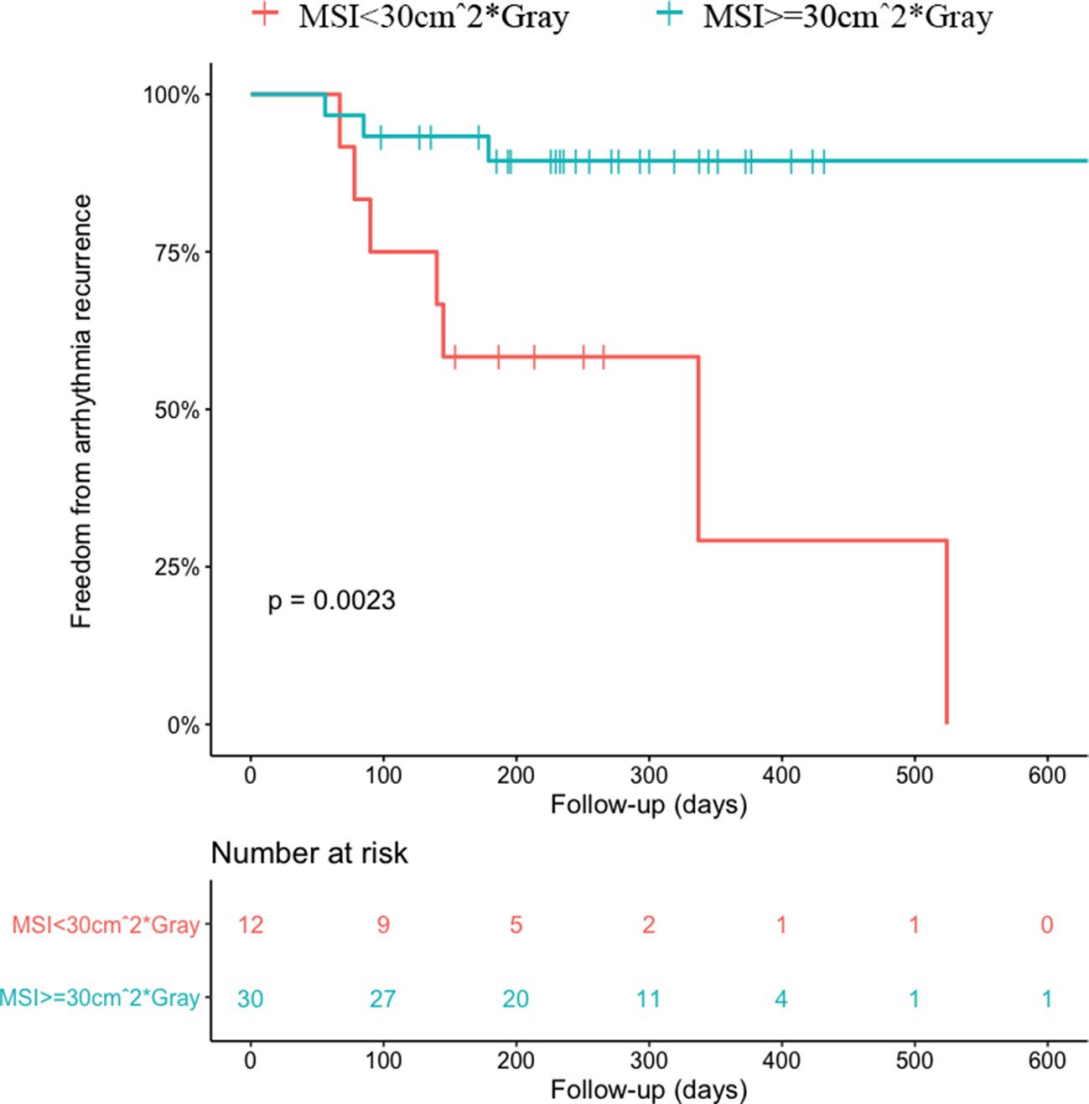
Kaplan–Meier curves for arrhythmia recurrence at latest available follow-up stratified for MSI values. MSI, myocardial staining index

Both MSI and MI block achievements were significantly different between patients who had an arrhythmic recurrence, particularly in those who had a recurrence as atrial flutter (Table 3). Cox regression analysis revealed that MSI was associated with atrial flutter recurrence (HR 0.95, 95% CI 0.91 – 0.99, *p* = 0.043), but not with overall arrhythmia recurrences (HR 0.98, 95% CI 0.95 – 1.01, *p* = 0.159). Of note, patients on AADs had lower MSI [36.2 (17.9 – 45.1)] than patients off AADs [51.3 (31.3 – 62.7)] (*p* = 0.033), but similar rates of MI block (*p* = 0.558). Cox regression analysis also showed a strong association between MI block and both arrhythmia recurrence (HR 0.11, 95% CI 0.03 – 0.41, *p* = 0.001) and recurrence as atrial flutter (HR 0.09, 95% CI 0.01 – 0.51, *p* = 0.007). AUC for the prediction of arrhythmia recurrences for MSI was 0.713 (Fig. 6). A cutoff of 30 cm^2^ × Gray for MSI was identified. Kaplan–Meier analysis based on the previously identified MSI cutoff showed a log-rank of 0.002 (Fig. 7).

**Table 3:** Arrhythmia recurrence

	Arrhythmia recurrence (*n* = 10)	No arrhythmia recurrence (*n* = 32)	*p*-value	Recurrence as atrial flutter (*n* = 6)	No recurrence as atrial flutter (n = 36)	*p*-value
MSI (cm^2^ × Gray)	24.7 (14.9 – 52.7)	45.4 (38.3 – 58.9)	0.045	22.2 (14.9 – 35.6)	45.5 (35.1 – 58.9)	0.027
MI block achieved	5 (14.3%)	30 (85.7%)	0.001	3 (8.6%)	32 (91.4%)	0.018
MI block not achieved	5 (71.4%)	2 (28.6%)		3 (42.9%)	4 (57.1%)	
AADs	2 (20.0%)	12 (37.5%)	0.306	2 (33.3%)	12 (33.3%)	1.000

*MSI*, myocardial staining index; *MI*, mitral isthmus; *AADs*, antiarrhythmic drugs

## Discussion

Myocardial contrast staining occurring during VOM ethanol infusion can be quantitatively assessed by a myocardial staining index (MSI). In our study cohort, a higher MSI predicted mitral isthmus block occurrence and a reduced time and number of RF applications, and was significatively related to the possibility to obtain it with just endocardial ablation, sparing the need of additional RF applications into the coronary sinus.

### Global and localized myocardial staining

Selective venography performed during VOM-EI shows staining of atrial myocardium resulting from retrograde contrast medium diffusion through damaged capillaries. When ethanol exerts its effect over the whole VOM-associated capillaries, a global MS can be observed. Differently, localized MS may sometimes occur because of vascular dissection due to balloon inflation, guidewire and sheath manipulation, or high-pressure infusion, potentially limiting adequate spreading of ethanol in the tissue [20]. Therefore, localized MS can be visualized as discrete leakage of contrast medium, in addition to variable grades of global MS, spreading concentrically from the point of vascular injury. Localized MS may induce the operators to discontinue the procedure to avoid potential complications such as pericardial effusion and cardiac tamponade [22].

**Fig. 8:**
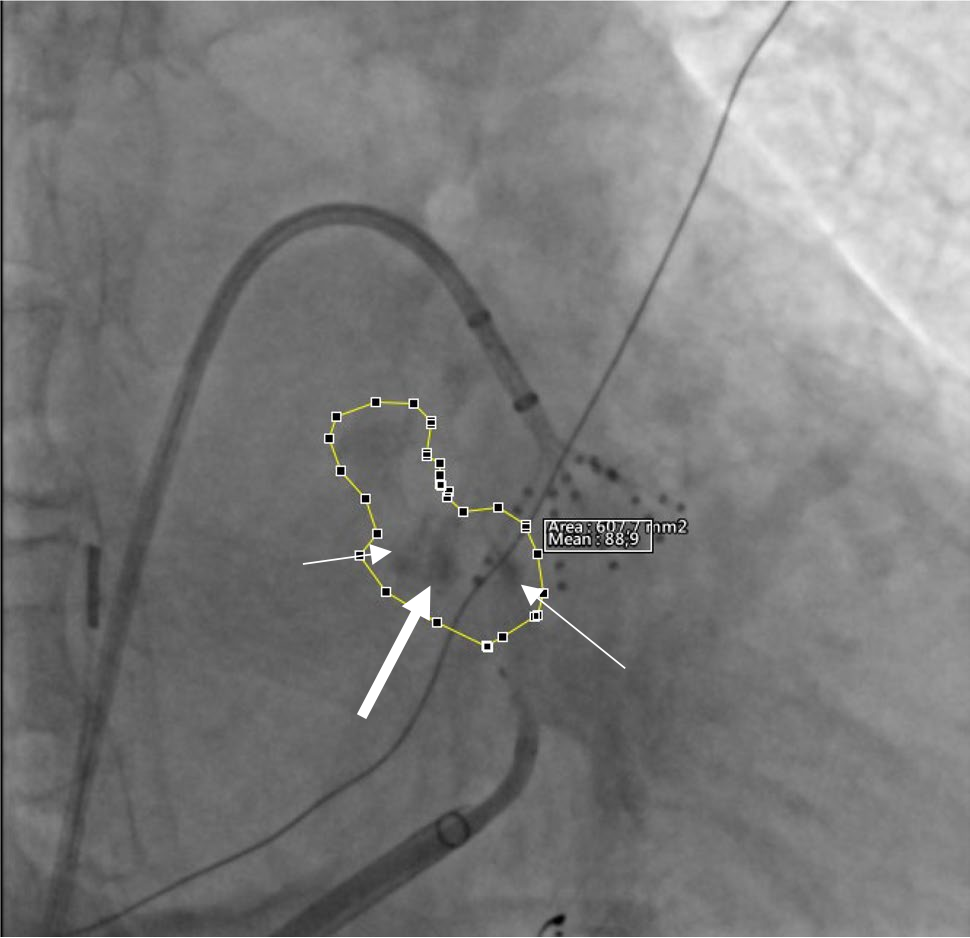
Concomitant localized myocardial staining (arrows) and global myocardial staining (polygon)

In a previous study by Takagi et al., localized MS did not impact ethanol-induced lesions evaluated through size of endocardial LVZs nor the achievement of acute MI block [20]. However, the analysis was limited by the dichotomic qualitative grouping of the study population for the occurrence of localized MS, not accounting for the various degrees of concomitant global MS (Fig. 8). Indeed, localized MS occurrence does not exclude the possibility of various extension of simultaneous global MS, which should be considered the main responsible for the observed ethanol-induced lesion. As such, relying only on the presence or absence of localized MS during VOM-EI procedures may be misleading.

### Myocardial staining index

We proposed MSI as a quantitative tool to assess MS, whether global or localized or both. MSI accounts for both the extension and the intensity of MS, enclosing under a single variable a comprehensive assessment of the ethanol-induced lesion. The advantage of relating the extension of MS to the delta fluorographic luminescence is to correct for the fixed angle of fluorographic acquisition. The defined angle of acquisition may not be orthogonal to the MI surface in all patients indeed; therefore, in these cases, a smaller area would be corrected by an increased opacity due to the superimposition of different planes. Also, considering the delta fluorographic luminescence (i.e., difference between pre- and post-VOM-EI fluorographic luminescence) allows to take into account only the additional opacity derived from the VOM-EI procedure, avoiding potential errors derived from superimposition of cables and/or catheters. As a confirmation of the goodness of the measure, MSI demonstrated a high correlation with the delta LVZs provided by VOM-EI. While MSI represents a direct index of ethanol-induced lesion, delta LVZs is an indirect one as a measure of the final effect on the LA myocardium.

### Association and prediction of mitral isthmus block

In our study cohort, both delta LVZs and MSI were significantly associated with MI block occurrence and were able to predict it with high accuracy. It is known that a good VOM-EI procedure results in higher rates of MI block or, conversely, that MI block is expected to occur more frequently when VOM-EI is satisfying. Importantly, it is not the presence or absence of localized MS that should be looked after, but the concomitant global MS, as this is the main determinant of the ethanol-induced lesion.

Moreover, in our patient population, MSI was significantly associated not only with the occurrence of MI block, but also with its achievement by endocardial ablation only. A high MSI seems to suggest a homogeneous spread of the ethanol over the epicardial MI surface, potentially leaving only a small endocardial layer intact, more frequently on the ventricular side of the MI. This observation might be explained by the presence of the “blind corner” at the ventricular side provided by the VOM segment which hosts the inflated angioplasty balloon. Therefore, it is not surprising that a higher MSI was associated also with a reduced ablation time and reduced RF applications to obtain MI block.

### Association with arrhythmia recurrence

Overall, the arrhythmia recurrence was similar to the one previously reported in a study with the same treatment approach, even though rate of AADs administration was higher in our study [19]. Even though recent evidence suggests revisiting the definition used for arrhythmia recurrence [23], having a cutoff of 30 s is probably insignificant from a clinical point of view as compared to arrhythmic burden reduction, for instance, we decided to keep this definition in order to be as sensitive as possible. However, despite the raw numbers of arrhythmia recurrences, analysis of those patients with an ILR/CIED revealed a low arrhythmic burden and duration of recurrence episodes. Recurrences were almost equally represented by AF and atrial flutter. As expected, patients with a recurrence as atrial flutter had lower MSI and lower rate of MI block at index procedure. This was true for overall arrhythmia recurrence as well, but association was not confirmed for MSI. However, patients on AADs had lower MSI, probably as a result of the perceived ineffectiveness of VOM-EI by the operator who preferred AAD continuation beyond the blanking period, potentially limiting recurrence rates in this subgroup.

### Clinical implications and study limitations

We have long observed that the more MS occurs during VOM-EI procedures, the easier MI block is reached. Such observation has initially led us to aim for a satisfactory degree of visually assessed MS during VOM-EI procedures to improve success rates. However, even though MS is an easy interpretable phenomenon, we wanted to find a measure to objectively quantify it and verify our hypothesis. Therefore, after many attempts to enclose under a single variable a reliable reflection of ethanol-induced lesions, we came out with the presented MSI. Different from visually assessed MS, MSI has the downside of being less immediate but is much more reproducible. Moreover, time to MSI calculation should be of limited concern with a properly set workstation. Hence, the impact of the pause required for MSI assessment on the overall duration of procedure should be very small. Besides, we also observed that delta LVZs could be used as a measure of effectiveness of VOM-EI as well, but it is limited by that fact that it can be assessed only at remapping, when VOM-EI is already finished, therefore resulting less useful in contrast to MS.

MSI could support the intraprocedural assessment of MS as a measure of efficiency and effectiveness of VOM-EI and potentially set a tailored ablation strategy. For instance, it could lead the operator to optimize balloon position and/or inflation, thus reducing ethanol leakage, or limit alcohol delivery in case of sufficient MS with lower than standard doses. Such fine adjustments could also improve safety besides efficiency. In a recent series from Leyton-Mange et al. [24], the overall complication rate of VOM-EI was more than 5%, of which delayed tamponade represented around 3%. In our experience, an overall higher rate of complications occurred, even though no one resulted in tamponade. In particular, the most observed complication, VOM perforation, never resulted in serious complications. In our opinion, the higher rate of VOM perforation as compared to that reported by the Bordeaux group (2.8% vs 7.1%) could be explained by the higher experience of that center in VOM-EI procedure [25]. Indeed, our procedural protocol is derived from theirs, which includes selective VOM angiogram repeated each time after ethanol bolus injection to confirm balloon stability; therefore, the multiple injections protocol should not be responsible for this observation [25].

To conclude, evidence emerging from this study could help the operators (1) to estimate the acute impact of VOM-EI, potentially justifying a tailored amount of alcohol infused and more extensive endocardial/GCV RF applications; and (2) to assess the correct delivery of alcohol into the VOM, alerting for possible CS/atrial/pericardial drainage.

This study has some important limitations, such as the small number of patients in the study population, the inclusion of both naïve patients and redo cases, the different means of investigation used during follow-up, and the unstandardized AAD regimens. The analysis is also limited by the retrospective calculation of MSI, even though this process was carried out by a single operator blinded to procedural results. We suggest that MSI could potentially be used to guide VOM-EI assuming that MSI increases linearly with the amount of ethanol injected, which is an observation derived from our experience but for which we have not reported specific data in support of. Finally, we do not have prospective data on the correlation between MSI and durable scar formation. Results should therefore be interpreted in the context of these limitations.

## Conclusion

Bidirectional MI block is difficultly obtained even when VOM-EI is performed. Myocardial staining occurring during VOM-EI predicts MI block and is associated with its achievement with endocardial ablation only. Quantification of myocardial staining by MSI confirms its high correlation with newly formed LVZs.

## Data Availability

The data underlying this article will be shared on reasonable request to the corresponding author.

## Abbreviations

PVI: Pulmonary vein isolation
PeAF: Persistent atrial fibrillation
VOM: Vein of Marshall
EI: Ethanol infusion
MS: Myocardial staining
LVZ: Low-voltage zones
OTW: Over-the-wire
MSI: Myocardial staining index
MI: Mitral isthmus
LA: Left atrium
LAA: Left atrial appendage
CS: Coronary sinus
GCV: Great cardiac vein
LSI: Lesion size index
ROC: Receiver operator characteristic
AUC: Area under the curve
